# Is self-determination good for your effectiveness? A study of factors which influence performance within self-determination theory

**DOI:** 10.1371/journal.pone.0256558

**Published:** 2021-09-08

**Authors:** Michał Szulawski, Izabela Kaźmierczak, Monika Prusik

**Affiliations:** 1 Institute of Psychology, The Maria Grzegorzewska University, Warsaw, Poland; 2 Department of Psychology, The University of Warsaw, Warsaw, Poland; University of Hradec Kralove: Univerzita Hradec Kralove, CZECH REPUBLIC

## Abstract

Despite the vast body of studies within self-determination theory, the impact of factors which influence performance in experimental paradigm is still underresearched. The aim of the two studies presented in this paper was to investigate the impact of basic psychological needs on performance with the simultaneous presence of external incentives. Study 1 tested whether the satisfaction of competence and relatedness during task performance (while external incentives were present) can impact individual’s performance. Study 2, on the other hand, investigated whether the basic psychological needs and provision of external incentives can impact an individual’s performance. Moreover, in both studies the mechanisms behind the need–performance relationship was checked. Our results showed that out of the three basic needs, competence had the strongest positive impact on performance, which was partially mediated by the subjective evaluation of the levels of difficulty and intrinsic motivation. The weak relationship between relatedness and task performance was fully mediated by the level of intrinsic motivation.

## Introduction

When we asked people a question concerning the reasons why they work, learn, or exercise efficiently, with a high degree of probability they would enumerate some kind of incentives or rewards (remuneration, grades, or bonuses) at the top of their lists. This common belief has almost become an axiom, which can make people reject—or at least undermine—other possible explanations of what could increase the performance of their actions or undertakings. This explanation, often viewed with scepticism, of what (except incentives) can predict or enhance performance, is offered by self-determination theory (SDT); [1–3], which is one of the most often cited theories of human motivation. SDT depicts human beings as having three basic psychological needs which contribute to the development of their intrinsic motivation towards striving, well-being, and performance [4]. However, understanding of the influence of motivational factors within the theory, such as the three needs and incentives on performance has been mostly studied outside the laboratory contexts [5–8]. The three basic psychological needs of SDT, namely autonomy, competence, and relatedness, are defined as universal and relevant within all people and cultures [9]. Autonomy involves feeling internal approval of one’s behaviour, thoughts, and emotions rather than feeling controlled or pressured; competence involves feeling efficient and qualified in one’s behaviour, rather than incompetent and ineffective; and relatedness involves feeling meaningfully connected to others, rather than feeling alienated or ostracized. Over the past decades, researchers have established that satisfaction of autonomy, competence, and relatedness is critical to most domains of human functioning such as health care [10, 11], mental health [12, 13], the development of well-being [14, 15], the development of intrinsic motivation [16], engagement at work [17, 18], and in sports [19, 20] to name a few. However, the links between needs satisfaction and performance has not attracted much attention from researchers [6].

### Basic psychological needs and performance

The way in which autonomy, competence, and relatedness are understood in self-determination theory is quite unique in the performance context [6]. According to SDT, and in opposition to other needs theories, the three needs do not vary by the extent to which people possess them, but by the extent to which the environment facilitates their satisfaction or frustration [9]. The main reason for why the three needs could influence performance is that the environments which facilitate the needs build autonomous and intrinsic types of motivation, which consequently improves performance. Extensive research confirms the link between the needs satisfaction and autonomous motivation [2, 21–24] and between autonomous or intrinsic motivation and performance [25]. Moreover, recent meta-analysis confirms the relationship between basic needs and performance [6]. Authors also emphasise that there are other different reasons why they can predict performance, rather than an increase in internal motivation, which are exclusive for each of the needs. For the need for autonomy this is an internal locus of causality for the actions which lead to taking ownership of the action [6, 26]; for the need for competence it is the mix of challenge and skill which enables an individual to experience an action which is not too easy and not too difficult, and at the same time to possess skills which are necessary to do or accomplish the activity [20], and for the need for relatedness it is the well-being of the person during the performance of the action [27]. However, there is still little research which shows the mechanism of the needs–performance relationship in an experimental context, especially combined with the presence of external incentives. In one research study where all three needs were manipulated experimentally, only competence had a positive impact on performance [13]. It is worth noting that in most, if not all, environments, such as school, work, or academia, the provision of external incentives (grades, remuneration, rewards) usually coexist with the possibility of supporting or frustrating the three needs (the organization of work, leadership style etc.). As a consequence, it is worth studying simultaneously the influence of both the three needs and incentives on performance.

### Incentives and performance

In the context of self-determination theory, investigation of the relationship between incentives and performance has come to, what is called, an *uncomfortable conclusion*. In the body of research there is evidence for three seemingly true, but incompatible conclusions: (a) incentives boost performance, (b) intrinsic motivation boosts performance, and (c) incentives reduce intrinsic motivation (the undermining or crowding-out effect) [25]. Authors suggest that the uncomfortable conclusion is only seemingly true, as there was little research which took into consideration the situation where both incentives (extrinsic motivation) and basic psychological needs (intrinsic motivation) are present at the same time, and as there are possible different mechanisms (mediators) of the relationships.

When it comes to incentives and rewards, they are commonly regarded as an adequate means to improve performance [28, 29]. Studies concerning incentives usually confirm the positive influence of incentives on individual performance [30–33], especially in short term [34]. According to meta-analysis the effect sizes of individual incentives on performance ranged from.19 to.39 (for different moderators) with a general effect size of.34 for the individual incentives from 116 studies [35].

The idea of the undermining effect (or crowding-out effect) also has a long tradition of research in the paradigm of Cognitive Evaluation Theory (CET, a mini-theory of Self-Determination Theory) [24]. The studies have proven that rewards (alongside punishments and deadlines, for instance) may undermine intrinsic motivation towards a given activity by thwarting the need for autonomy (people perceive the reason as to why they do an activity as controlled and not chosen by them) and for competence (the focus is not on learning the activity but on the salient reason for doing it, e.g. a reward) [36–39]. Intrinsic motivation is understood as a ’spontaneous, evolved and inherent propensity (…) to develop through activity, to play, explore, and manipulate things, and by doing so develop competencies and capacities’ [24, p.123].

In two studies we tried to investigate the simultaneous impact of incentives and basic psychological needs on performance.

### Current study

#### Study 1

The aim of the first study was to test whether the satisfaction of two out of three basic needs,–competence (competence supported or not) and relatedness (relatedness supported or not) during task performance and while external incentives were present can impact individual performance. The predictions of the possible outcomes were based mostly on the meta-analysis of the simultaneous impact of incentives and basic psychological needs on performance [6], experimental studies on influence of needs on performance [13] and the assumptions of self-determination theory [3]. It was suspected that, even though the external incentives were present and salient during the activity and might reduce intrinsic motivation [39, 40], support for the need for competence and relatedness should positively influence performance. The meta-analysis showed a stronger relationship between competence and performance [6], and the experimental study conducted by Sheldon and Filak [13] showed that only competence (out of the three basic needs) had a positive impact on performance in the game-playing context. The groups with supported competence need should evaluate the task as less challenging, and the perception of a task which was not difficult should result in higher performance [41]. As a consequence, the relationship between support of the competence need and performance should be mediated by the level of intrinsic motivation and the level of subjective challenge of the task. Supporting the need for competence should result in a higher intrinsic motivation for doing the task, and as a consequence, higher motivation should result in higher performance. Supporting the need for relatedness, on the other hand, should also result in higher intrinsic motivation for doing the task, and higher motivation should result in higher performance [24]. The support of the need for relatedness should also increase affective well-being, which may have a positive impact on performance. Non-experimental studies suggest that well-being is related to performance [42, 43].

Based on the aforementioned theoretical considerations and empirical research, the following three hypotheses were formulated:

H1: Groups with supported basic psychological needs (competence, relatedness) will have a higher level of task performance.
H2: The relationship between support of the need for competence and task performance level will be mediated by the level of intrinsic motivation and subjective challenge.
H3: The relationship between the support of the need for relatedness and task performance level will be mediated by the level of intrinsic motivation and affective well-being.

In the first study participants received remuneration in all conditions for each of the origami figures folded, so the external incentives were present in all conditions. Even though, study 1 has no comparison group without incentives, the conditions in the study reflect the natural job conditions where people receive remuneration for their efforts, and still the environment may be more or less need-supportive.

#### Study 2

Study 2 examined whether the basic psychological needs (the three needs: supported vs. frustrated) and provision of external incentives (whether rewards were present or not) can impact the individual’s performance. The predictions and hypotheses were again based on the meta-analysis conducted by Cerasoli [6] and the assumptions of self-determination theory [3]. It was assumed that provisions of incentives and support of basic psychological needs would positively impact performance. Out of four experimental groups, the group with both external incentives present and with their basic psychological needs supported would have the highest performance. We posited that even though the incentives in Study 2 are salient (so they can undermine intrinsic motivation) they would have a direct positive impact on performance. The needs–performance relationship would be mediated by intrinsic motivation and by the self-reported measure of the three basic psychological needs.

Three hypotheses were formulated:

H4: Groups with supported basic psychological needs will have a higher task performance level than the groups without support.
H5: Groups in which individuals are granted external incentives will have a higher task performance level than the groups without incentives.
H6: The relationship between the needs support and task performance level will be mediated by the level of intrinsic motivation and level of basic psychological needs (self-report).

## Method

### Study 1

#### Participants

One hundred and eight university students (*N* = 54 females) from different Varsavian Universities and different faculties (e.g. Educational Studies, Sociology, IT, German and English Philology) aged 19–33 years (*M* = 23.03, *SD* = 2.96), participated in the study. The protocol of the study was approved by the University’s Ethical Committee. All participants gave written informed consent in accordance with the Declaration of Helsinki before participation.

#### Instruments

**Task performance level** was the number of origami figures folded by the participant which ranged from 0 to 10.

**Subjective difficulty** was measured by a 3-item declarative 10-point scale. The items were as follows:

*’How difficult will the task of folding origami be for you*?*’* (1—very easy; 10—very difficult)
*’What are your chances of folding the origami figures well*?*’* (1—a very small chance; 10—a very high probability, verging on certainty)
*’How much mental effort will folding origami be for you*?*”* (1—very little effort; 10—very high effort)

**Intrinsic motivation** related to laboratory activity was measured with the Intrinsic Motivation Inventory (IMI) [3, 44]. The interest/enjoyment subscale is considered the self-report measure of intrinsic motivation; thus, although the overall questionnaire is called the Intrinsic Motivation Inventory, it is only the one subscale that assesses intrinsic motivation. The subscales have good psychometric properties, with the interest/enjoyment alpha α = .78.

**Affective well-being** was measured with Positive and Negative Affect Schedule (PANAS); [45]. The short, 20-item, state version of the scale was used. The scale has good psychometric properties, with an alpha α = .80 (positive emotion scale) and.91 (negative emotion scale). Such a measure of well-being was previously used in research on well-being [46–48].

In the study 1, the level of wellbeing was measured with the PWB questionnaire [49]. The index of wellbeing was not used in the study, it was measured for the purpose of future, follow-up study.

#### Procedure

First, the participants were told that they would be taking part in a study about the use of origami in research on learning, which would involve folding simple origami puzzles. Participants who agreed to take part signed in through a computer platform to come for a meeting at a specific date and time. The experiment took place during one, individual meeting in a research room (this was the same for all participants) and lasted about 40 minutes for each participant. The experimenter explained that the participants would be folding origami puzzles and would receive 2 PLN (approx..50 EUR) for each origami figure folded. The participants were told that they could fold as many figures as they liked and after each figure had been folded the experimenter would ask them whether they wanted to fold another figure. The experimenter also explained that the participants would fill in a few questionnaires at the end of the meeting. Next, the experimenter showed the participant an example of a simple origami instruction and asked them to evaluate how difficult/challenging the task of origami folding would be for them (see subjective challenge measure). Each participant was then randomly assigned to one of the four experimental groups according to 2 (competence supported or not) x 2 (relatedness supported or not) experimental design.

The groups differed in the way the experimenter gave instructions on how to fold origami, how he commented on the results, and what he did with the origami which was folded (see needs manipulation procedure below). The rest of the procedure was the same for all the groups. After evaluating the subjective challenge, the experimenter asked the participants whether they wanted to fold the first origami, and if they agreed he gave them the choice of three origami figures to fold; the participant chose one and folded it. Then, the procedure was repeated until the participant said that they did not want to fold any more figures or they reached 10 figures folded (see performance measure). The time for folding origami was not limited, it took on average two to three minutes to fold each origami piece. After that the experimenter asked the participant to fill in a few questionnaires. Then the participant was paid for the number of puzzles they had decided to fold and was asked what they thought about the study and whether they had any questions. Then they were debriefed and thanked.

*Needs manipulation procedure—competence*. In the groups with enhanced competence need the experimenter gave the participants some short feedback after each of the first three origami puzzles had been folded and then after every second folded figure. The feedback consisted of short comments concerning the way the person folded the origami, for example: ’You’ve done it very skilfully’ or ’You’ve managed to put it together quickly’.

In the groups with no competence enhancement the experimenter gave a short comment after each of the first three origami puzzles had been folded and then after every second folded figure. The comment only concerned the general process of the experiment, for example: ’Ok, thank you’ or ’Ok, we will put it here’, so that the communication process was sustained but without any feedback on the competence.

*Needs manipulation procedure—relatedness*. In the groups with enhanced relatedness need, the experimenter asked the participants whether he / she could use the folded origami for a workshop he was holding with children the other day, and if the participant agreed, he put the folded origami into the box entitled ’materials for workshop’. All the further origami folded by the participant was also put into the box without any further comment. In the group with no relatedness enhancement the experimenter put all the figures folded by the participant into the box without an inscription and without any further comment.

#### Data analysis

Statistical analyses were performed using IBM SPSS Statistics 26 software (IBM Corporation, 2019) to compute ANOVA analysis, and Hayes PROCESS Macro v3.5 to compute mediation analyses.

### Results

To test the first hypothesis (H1) which was related to the role of competence and relatedness enhancement on task performance level, we ran bootstrapped two-way between-subjects ANOVA (Table 1). There were significant main effects of competence and relatedness according to which enhancement of either competence or relatedness resulted in better task performance (a higher number of origami puzzles folded). Also, the combined effect (interaction) of these two factors was significant. An analysis of simple effects showed that out of four simple effects only one of them was not statistically significant. Within the group in which competence was not supported there was no significant difference (*p* = .956, BCa 95% CI [-1.27, 1.26] between relatedness supported (*M* = 4.52, *SE* = 0.48, *Bias* = .003, BCa 95% CI [3.68, 5.46]) or not supported (*M* = 4.56, *SE* = 0.48, *Bias* = .010, BCa 95% CI [3.54, 5.56]) on the number of origami puzzles folded. Whenever competence was supported there was a significant difference (*p* = .002, BCa 95% CI [-3.23, -0.95]) between relatedness not supported (*M* = 5.96, *SE* = 0.48, *Bias* = -.015, BCa 95% CI [4.86, 7.07]) and supported (*M* = 8.04, *SE* = 0.48, *Bias* = .017, BCa 95% CI [7.22, 8.84]). In the group in which relatedness was not supported there was a significant difference at statistical tendency (*p* = .060, BCa 95% CI [-2.89, 0.13]) between situations in which competence was not supported (*M* = 4.52, *SE* = 0.48, *Bias* = .003, BCa 95% CI [3.68, 5.46]) or when competence was supported (*M* = 5.96, *SE* = 0.48, *Bias* = -.015, BCa 95% CI [4.86, 7.07]). Also, when relatedness was supported there was a significant difference (*p* < .001, BCa 95% CI [-4.80, -2.22]) between a situation in which competence was not supported (*M* = 4.56, *SE* = 0.48, *Bias* = .010, BCa 95% CI [3.54, 5.56]) in contrast to competence being supported (*M* = 8.04, *SE* = 0.48, *Bias* = .017, BCa 95% CI [7.22, 8.84]). In summary, the highest number of origami puzzles folded occurred in groups in which both competence and relatedness were supported. However, support of any of these needs resulted in better task competition, with the role of competence supported definitely being more important than the role of relatedness. Thus, H1 was entirely confirmed.

**Table 1:** Two-way between-subjects bootstrapped ANOVA for task performance level in Study 1.

	Non-supported					Supported								
	*M*	Bias	*SE*	5%, BC_a_ CI	95%, BC_a_ CI	*M*	Bias	*SE*	5%, BC_a_ CI	95%, BCa CI	*F*	*df*	*p*	η_p_^2^
**Competence**	4.54	.02	.33	3.86	5.27	7.00	.01	.37	6.34	7.70	26.31	1, 104	< .001	0.20
**Relatedness**	5.24	.01	.36	4.53	5.98	6.30	.02	.39	5.47	7.12	4.83	1, 104	.030	0.04
**Interaction**	-	-	-	-	-	-	-	-	-	-	4.50	1, 104	.036	0.04

N = 108, bootstrap = 2000.

**Fig 1:**
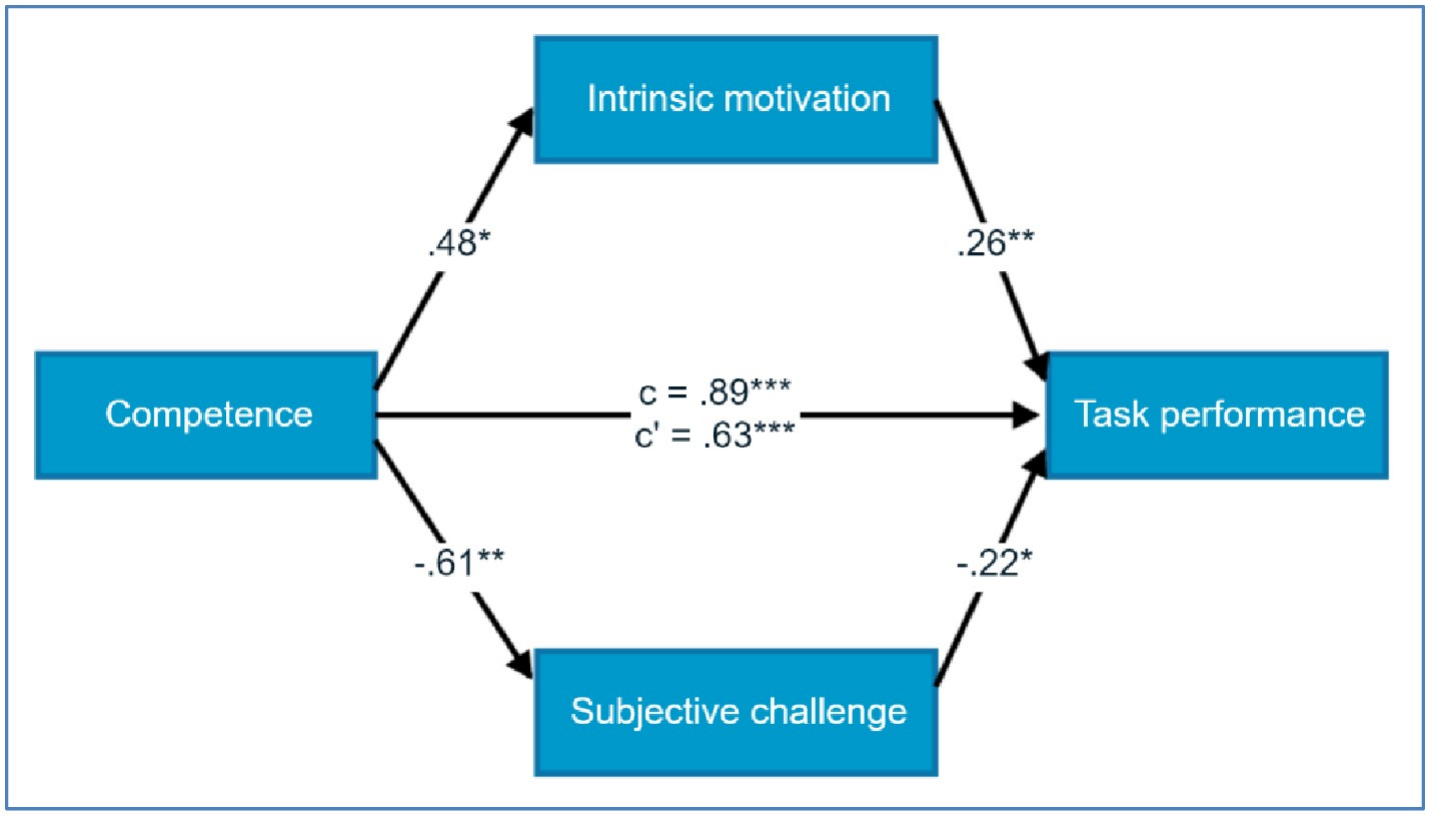
Standardised regression coefficients for the mediational effects of intrinsic motivation and subjective challenge in the relationship of competence and task performance level in Study 1.

Mediational hypotheses (H2 and H3) were tested using Model 4 in Process ver. 3.5 by Hayes (2018). Allied with H2, the relationship between the support of the need for competence and performance level was partly mediated by both intrinsic motivation and subjective challenge taken together as parallel mediators (overall indirect effect), *ab* = 0.74, BCa 95% CI [0.29, 1.28], and also separately by subjective challenge (particular indirect effect), *ab* = 0.38, BCa 95% CI [0.05, 0.82], and by intrinsic motivation (particular indirect effect), *ab* = 0.36, BCa 95% CI [0.06, 0.74] (Fig 1). In summary, H2 was confirmed.

**Fig 2:**
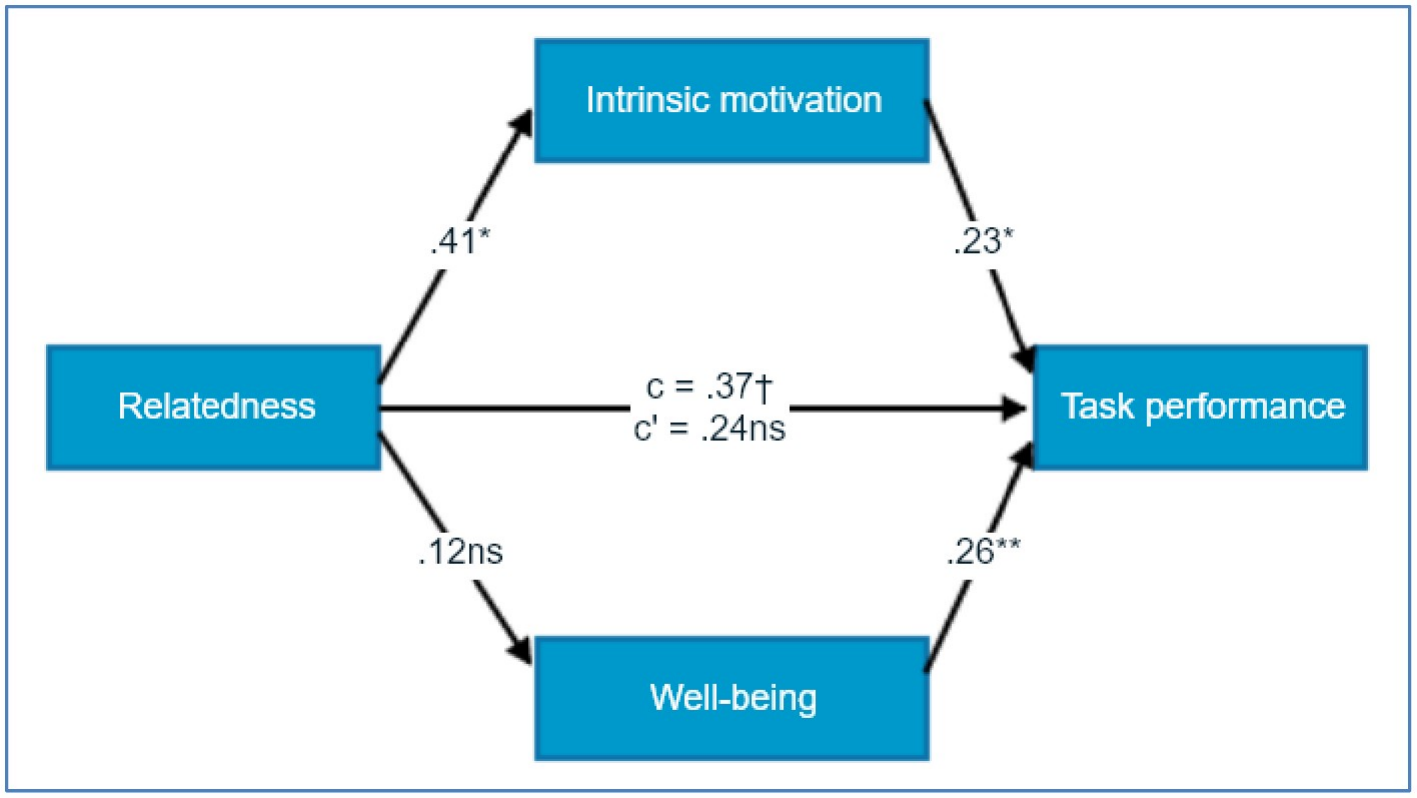
Standardised regression coefficients for the mediational effects of intrinsic motivation and well-being in the relationship of competence and task performance level in Study 1.

According to the testing of H3, the mediational effects both for the overall effect of combined mediators (overall indirect effect), *ab* = 0.36, BCa 95% CI [-0.11, 0.90], and well-being, *ab* = 0.11, BCa 95% CI [-0.24, 0.49] were not confirmed. However, there was a significant mediational effect of intrinsic motivation, *ab* = 0.127, BCa 95% CI [0.01, 0.63]. Thus, H3 was only partly supported by our results (Fig 2).

### Discussion

In the first study we attempted to investigate the influence of two of the basic psychological needs (competence and relatedness) on performance. Furthermore, we tried to study the mechanisms of the relationship between the basic needs and performance. A novel and important element of this study was the coexistence of context factors which satisfied the needs with the simultaneous presence of salient external incentives.

In general, the results were in line with Hypothesis 1, which predicted that even with the presence of external incentives, the basic psychological needs impact performance. The positive impact of the need for competence was stronger than that of the relatedness, which corroborates the meta-analysis results in which the overall influence of competence was the strongest of all three needs [6] and the Sheldon and Filak’s experiment, where only competence predicted performance [13]. It is worth noting that the rewards received by the participants for folding origami puzzles were salient. The previous studies suggest that the overall need for satisfaction is lower when salient incentives are present [6]. This could be explained by the undermining effect lowering autonomous or intrinsic motivation for the task, when direct, easy to notice and understand incentives are present [37, 50]. Still, the result of our study suggests that even when external rewards are present and salient, satisfaction of the needs may have a positive influence on performance.

The second hypothesis, stating that the relation between the need for competence and task performance is mediated by subjective evaluation of the level of difficulty (subjective challenge) and by the level of intrinsic motivation, has also been confirmed. Indeed, the influence of competence on performance was pa2rtially explained by the subjectively lower challenge of the task (making the task seemingly easier) which further resulted in higher performance. We believe that by giving positive feedback on participants’ competence, the new task they were doing was perceived as easier and as a consequence closer to their optimal level of challenge. Studies suggest that the challenge which is perceived as optimal (not too easy and not too difficult) is positively related to performance [20, 51, 52]. The second mediator, intrinsic motivation, explained the relation between competence and performance to a lesser extent (statistical tendency). According to SDT, intrinsic motivation is related to performance, especially in the qualitative task [25]. However, it is again worth noting that the results were achieved even with the presence of external rewards. Finally, both mediators explained only part of the relation, and the direct effect of competence on performance persisted, which may be explained by the rise of self-efficacy, which is a well-evidenced antecedent of performance, but which we did not measure in our study [41].

Hypothesis 3, stating that the relation between relatedness support and performance level will be mediated by the level of intrinsic motivation and well-being, has only been partially confirmed. Firstly, the influence of relatedness on performance was not strong (statistical tendency), and the relation was mediated inclusively and fully by the level of intrinsic motivation, but not by the level of well-being. The rise of intrinsic motivation, and as a consequence slightly better performance due to relatedness need support, is in line with the assumptions of the SDT [3]. The meta-analysis also showed that relatedness has a much smaller influence overall on performance than competence, which seems to be true also when the external incentives are present [6]. The lack of relation between the relatedness and well-being may be connected with the way the relatedness was manipulated in this study. Contrary to other studies, the relatedness was enhanced not by the ’good rapport’ between the experimenter and the participant [13], but by the promise of a socially useful purpose for the origami puzzles created during the experiment.

## Study 2

### Methods

#### Participants

Two hundred young adults (*N* = 119 females) took part in the study. The participants were students (on courses such as Educational Studies, Sociology, Engineering, English Philology, and Philosophy) and employees from different companies (e.g. recruitment staff, IT workers, teachers) aged 18–34 years (*M* = 21.67, *SD* = 2.59). The protocol of the study was approved by the University’s Ethical Committee. Before participation all participants gave written informed consent in accordance with the Declaration of Helsinki.

#### Instruments

**Task performance** was measured by the sum of words found while playing Boggle in the first and second trials.

**Intrinsic motivation** related to laboratory activity was measured with the Intrinsic Motivation Inventory (IMI) [3, 44]. The interest/enjoyment subscale is considered the self-report measure of intrinsic motivation; thus, although the overall questionnaire is called the Intrinsic Motivation Inventory, it is only the one subscale that assesses intrinsic motivation. The subscales have good psychometric properties, with the interest/enjoyment alpha α = .78.

**Basic psychological** were assessed with the scale used by Sheldon for the experiment with needs manipulation [13]. The scale comprises of 9 items, three for each of the need. All items were administered with a 1 (strongly disagree) to 5 (strongly agree) scale. The subscales have robust psychometric properties with alpha α = .70 (autonomy), α = .80 (competence), α = .81 (relatedness).

#### Procedure

First, the participants were told that they would be taking part in a study about the use of games in research on learning, which would involve playing some kind of board game. Participants who agreed to take part signed in through a computer platform to come for a meeting at a specific date and time. The experiment took place in an individual meeting in a research room (this was the same for all participants) and lasted about 30 minutes for each participant. After entering the research room, the participants were informed that during the meeting they were going to learn how to play the game Boggle and fill in a few questionnaires. Furthermore, they were provided with informed consent with information about the anonymity of the results and voluntary participation. Each participant was then randomly assigned to one of the four groups align with 2 (reward / no reward) x 2 (needs supported vs needs frustrated) experiment design.

The groups differed in the way the experimenter gave instructions on how to play Boggle, how he commented on the results, and whether the participant received a reward for good performance (see the needs and reward manipulation procedures described below). The rest of the procedure was the same for all the groups and described below. The experimenter explained the rules of how to play Boggle to the participants, and that the higher the number of words the participant found, the better (see performance measurement). Then the participants played Boggle for the first time (the trial play) and subsequently a second time. The time for each player’s turn was limited to 3 minutes, as in the original rules of the game. After playing Boggle participants filled in the three questionnaires—the Intrinsic Motivation Questionnaire, and the Sheldon Needs Measure. Then the participants were asked what they thought about the study and whether they had any questions, and they were debriefed and thanked.

*Reward manipulation procedure*. After signing the informed consent in the groups where rewards were given to the participants, they were told that if they performed well during the Boggle game (i.e. they found more words than the average participant) they would receive a 45 PLN (approx. 10 EUR) voucher which could be used in the local grocery shop. At the end of the research, after filling in the questionnaires and before the debrief, all the participants from the reward group received the vouchers (they were told that they performed well, no matter if it was true or not). In the groups without reward the participants simply received information that the main rule of Boggle is that the more words you find the better.

*Needs manipulation procedure (adjusted from Sheldon & Filak* [13]*)*. In the groups where the **needs were enhanced**, the instructions on how to play Boggle and the results of each turn were modified so that the three basic psychological needs were supported. The examples before the first game were as follows.

*Before you begin, I would like you to remember that*:

1. *People who start playing the game usually don’t manage to find too many words*. *Try to do your best*, *you will learn quickly*, *I have confidence in your ability (competence)*.
2. *I would like you to know that you and your style of playing is important for us*. *Try to remember what kind of strategies you used during the game so we can talk about it later and use it as advice for other players (relatedness)*.
3. *I would like you to play this game in your own way*, *try to get to know this game and play with it*. *You can choose one of the three matrices (autonomy)*.

The instructions between the first and second game were:

*You quickly figured out how to play*, *you did well (competence)*.
*Remember that you and your individual style of playing is important*, *try to remember your playing strategies for future participants (relatedness)*.
*You can play this game using many different approaches*, *try to find those which suit you the best*. *Choose one of the matrices (three options) and tell me when you are ready to start (autonomy)*.

In the groups with **needs deprived** the instructions on how to play Boggle and the results of each turn were modified so that the three basic psychological needs were frustrated. The examples before the first game were as follows.

*Before you begin, I would like you to remember that*:

1. *People who start playing the game usually don’t manage to find too many words*, *but maybe you will be lucky (competence)*.
2. *In this study we are only interested in the group results as whole*. *Keep your observations to yourself as we go through the procedure (relatedness)*.
3. *In this experiment you have to proceed exactly according to the instructions which experimenter will give you and play according to the rules*. *You will start with this matrix (no choice of matrix given) (autonomy)*.

The instructions given between the first and second game were:

1. *Beginner’s luck or you weren’t lucky this time* (no matter if the participant succeeded or not, luck instead of competence is emphasised—competence).
2. (Not asking about the impressions or individual observations when such questions or comments are expected after the first trial game—relatedness).
3. *In the next game you will find words in this matrix*. *The time starts now* (no matrix choice was given, and the participant started when the experimenter asked him/her to do so—autonomy).

#### Data analysis

Statistical analyses were performed using IBM SPSS Statistics 26 software (IBM Corporation, 2019) to compute ANOVA analysis, and Hayes PROCESS Macro v3.5 to compute mediation analyses.

### Results

Again, we started the analytical work with two-way between subjects bootstrapped ANOVA to test the first hypothesis in Study 2 (H4). Results are presented in Table 2. The interaction effect of external incentives and needs supported was not significant, neither was the main effect of external incentives. The main effect of needs supported was significant and it indicated that the group with needs supported presented significantly better in task performance in comparison to the group without needs supported. This confirms H4 but does not confirm H5.

**Table 2:** Two-way between-subjects bootstrapped ANOVA for task performance level in Study 2.

	Non-supported					Supported								
	*M*	Bias	*SE*	5%, BC_a_ CI	95%, BC_a_ CI	*M*	Bias	*SE*	5%, BC_a_ CI	95%, BCa CI	*F*	*df*	*p*	η_p_^2^
**External incentives**	14.76	0.00	0.54	13.71	15.78	15.02	-0.01	0.49	14.12	15.91	0.15	1, 196	.699	0.00
**Needs supported**	13.83	0.01	0.50	12.86	14.85	15.88	-0.01	0.51	14.97	16.83	8.05	1, 196	.005	0.04
**Interaction**	-	-	-	-	-	-	-	-	-	-	1.54	1, 196	.217	0.01

N = 200, bootstrap = 2000.

**Fig 3:**
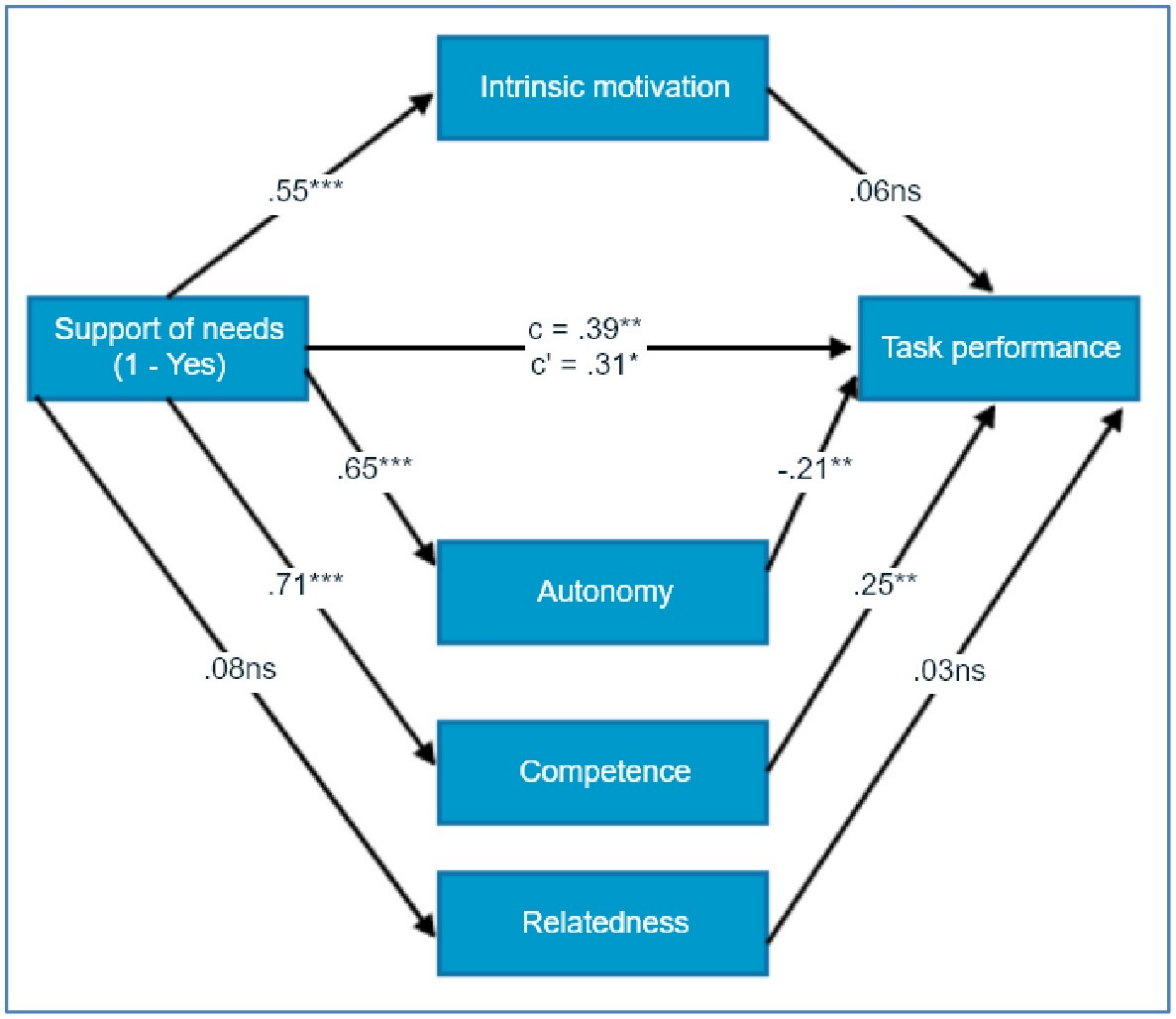
Standardised regression coefficients for the mediational effects of intrinsic motivation, autonomy, competence, and relatedness in the relationship of supported needs and task performance in Study 2.

Based on mediational analysis with the four parallel mediators (intrinsic motivation, autonomy, competence, relatedness), H6 was partly confirmed (Fig 3). The overall total effect was not significant, *ab* = 0.38, BCa 95% CI [-0.40, 1.22], as well a particular indirect effect for: intrinsic motivation, *ab* = 0.17, BCa 95% CI [-0.31, 0.71], autonomy, *ab* = -0.69, BCa 95% CI [-1.31, -0.14], relatedness, *ab* = 0.01, BCa 95% CI [-0.13, 0.16], except for a significant indirect effect for competence, *ab* = 0.89, BCa 95% CI [0.24, 1.63] (Fig 3). Competence was a significant mediator of the relationship between needs support and task performance. In conclusion, H6 was only partly confirmed.

### Discussion

In Study 2 we investigated the influence of the basic psychological needs (a context which either supported or frustrated the needs) and rewards on performance (which were either present or absent). Furthermore, we studied the mechanisms of the needs–performance relationship. A novel and important element of this study was the coexistence of context factors which satisfied the needs, with the simultaneous presence of salient external incentives. The results confirmed Hypothesis 4 which stated that supported basic psychological needs will have a higher level of performance in the accomplished task than the groups without support. The satisfaction of needs corroborates the results of studies where needs increase performance [6]. However, our further mediational analysis, which was a consequence of verifying Hypothesis 6, and which stated that the relationship between the needs support and performance level will be mediated by the level of intrinsic motivation and the self-reported level of basic psychological needs, was only partially confirmed. The analysis showed that the context which supported the needs during the experiment worked only on the needs of competence and autonomy, and out of these two needs only competence had a positive impact on performance and the need for autonomy, to our surprise, had a negative effect on performance. The meta-analysis suggests that the satisfaction of all three needs should have a positive impact on performance, with competence being its strongest predictor [6]. However, in the experiment conducted by [13] out of three needs only competence was a strong predictor of performance. It is especially worth noting, as in the current study, both the elements of the manipulation and the measure of performance (the Boggle game) were adjusted from the Sheldon and Filak [13] study.

Some studies suggest that autonomy has no impact on performance. For example, although the implementation of autonomous workgroups in a manufacturing environment resulted in a higher and lasting job satisfaction for employees, it had no consequences for work performance [53]. The findings of Saragih [54] can explain this. She showed that it is the self-efficacy that mediated the relationship between job autonomy and job performance. The question is whether enhancing the autonomy need might lead to a deterioration of the self-efficacy in certain situations (for example during performing a new task) and negatively impact performance. Moreover [55], found that the need for autonomy is associated with an avoidance of restrictive environments (such as constraints and rules). This suggests that the motivating role of the autonomy might be limited to some conditions. We can ask what if the aim of the task is entertaining (to take part in a study, play a game) and time is limited? The participants of Study 2, in the enhanced needs condition, were encouraged to experiment with the game Boggle (as a part of autonomy support), which they were playing for the first time and where the game itself lasted for a short time (2x3 minutes), even if they enjoyed the ’given autonomy’ in playing, they might have a need for more instructional (and less autonomous) forms of interaction, and an explanation of how to play, in order to play it correctly [56]. On the other hand, the explanation may be different, for the new task, the participants might have not taken the ownership of the action, which authors of SDT suggest is crucial for the support of the need of autonomy. The participants of the study took part in a new activity in a new environment and they were looking at the experimenter to guide them, even though he or she wanted to provide them more freedom of choice [6, 8].

Similar explanation may be offered to explain why the manipulation did not affect the relatedness need. The relatedness need support instruction was based on emphasising the interest of the experimenter in the participants’ strategies of playing the game and their usefulness for other participants. However, giving participants autonomy during the game might have been perceived as leaving them without actual support (not telling them how to play a new game). Furthermore, the manipulation had a positive effect on intrinsic motivation, which is in line with the results. Nevertheless, the intrinsic motivation was not associated with higher performance. The participants of the study felt inherent enjoyment in playing the game, but this did not translate into performance. In other words, if participants had more time for autonomous experimentation (enhanced autonomy) and inherent enjoyment of playing the game (intrinsic motivation) with time their performance would probably be also positively affected [3].

Another hypothesis (H5) stating that individuals who were granted external incentives will have a higher level of performance than the groups without incentives in the accomplished task, was not confirmed by our study. The hypothesis was formulated based on both SDT, which states that incentives build external forms of motivation [3] and studies and meta-analyses of incentive–performance relationships from outside SDT [31, 35]. We suggest that the reward for good performance might have caused the participants to focus on the reward instead of on the game and caused them to feel the additional stress of trying to earn the reward. Such results were confirmed in some earlier studies [57–59]. As a consequence, the reward’s positive motivational influence was eliminated by the additional stress that it caused.

## General discussion and conclusions

In the series of two studies, we tried to investigate the simultaneous impact of incentives and basic psychological needs on performance. Both studies confirmed that the need for competence had the strongest positive influence on performance. Study 1 showed that support of competence can influence performance directly, as well as through the decreasing subjective difficulty of the task and through increasing intrinsic motivation. These results are in line with earlier studies which did not, however, include extrinsic incentives in the study design [6, 13]. The need for autonomy (which was investigated only in Study 2) had a negative impact on performance. We assume that we achieved this effect mainly due to the fact that participants were taking part in a new and short task, which perhaps required a less autonomous, and more instructional kind of support from the experimenter in order to increase the participants’ effectiveness, or more time for the participants [56]. Relatedness had a low positive impact on performance in Study 1 (fully mediated by intrinsic motivation) and no impact in Study 2. Out of the three needs, the meta-analysis showed that relatedness is the least correlated to performance [6], and the study by [13] showed no impact of relatedness on performance.

Summing up we can draw a few conclusions from the two conducted studies:

1. in the context of performance (even when the external incentives are present) competence is the strongest and most certain predictor of performance,
2. relatedness and autonomy need more study in order to check their impact on performance. It is worth studying autonomy in different types of tasks (especially comparing new vs. known tasks), and both autonomy and relatedness are worth studying with tasks which last longer than in our study,
3. in general, the impact of needs and incentives on performance are worth studying simultaneously, as this is how they co-exist in ’real life’ (i.e. outside the laboratory),
4. in educational, occupational and sports contexts, where external incentives are the basic motivators (grades, bonuses, rewards) it is worth to introduce some interventions which may increase the support of the competence need (trainings, feedback, coaching) in order to increase individual performance of students, employees or athletes. The increase of competence support may influence the performance to a greater extent than the support of other needs. It is worth noting however, that in our study we investigated the influence of needs during the task which was interesting (games, putting together origami) and involved some cognitive capabilities and the results may not apply to the context of more imitative or reproductive kind of work (e.g. endurance type of sports or working at the assembly line).

### Study limitations and further research suggestion

Although, in general, the results confirmed most of our expectations, more research in this field is still needed. The biggest limitation of Study 1 is its sample. This study should be replicated with a more diversified and bigger sample of participants. In Study 2 it would be beneficial to divide the needs supported group into three separate groups where three basic psychological needs would be manipulated separately.

## Supporting information

S1 Data (SAV)

S2 Data (SAV)
